# The gender pain gap in prehospital analgesia: the role of patient and provider gender – an analysis of 106,888 helicopter emergency medical service (HEMS) missions

**DOI:** 10.1186/s12916-026-05131-0

**Published:** 2026-08-06

**Authors:** Andrea Schmidt, Hans-Jörg Busch, Lilith Huebsch, Florian Reifferscheid, Jörg Braun, Sebastian Heinrich, Julian Ganter

**Affiliations:** 1 Department of Anesthesiology and Critical Care, Faculty of Medicine, Medical Center - University of Freiburg, University of Freiburg, Freiburg, Germany; 2 DRF Luftrettung German Air Rescue, Filderstadt, Germany; 3 Department of Emergency Medicine, Faculty of Medicine, Medical Center - University of Freiburg, University of Freiburg, Freiburg, Germany; 4 Department of Anesthesiology and Intensive Care Medicine, University Hospital Schleswig-Holstein, Campus Kiel, Kiel, Germany

**Keywords:** Emergency Medicine, Gender Medicine, Analgesia, Air Rescue Service

## Abstract

**Background:**

Pain management is a core ethical principle in emergency medicine, yet gender-related differences in analgesic treatment have been reported. This study examines whether patient and emergency physician gender influence prehospital pain management.

**Methods:**

This retrospective observational cohort study included all primary helicopter emergency medical service missions conducted by DRF Stiftung Luftrettung in Germany between January 2012 and June 2025. Adult patients (≥ 18 years) with a Glasgow Coma Scale score ≥ 11 and without airway management were analyzed. Pain severity was assessed using the Numeric Rating Scale (NRS). Patient characteristics, analgesic treatment, and emergency physician gender were analyzed using descriptive statistical methods. The study was approved by the Ethics Committee Freiburg (25-1272-S1, August 19th 2025).

**Results:**

A total of 106,888 cases was included, of which 38.5% were female and 61.5% male. Initial pain severity was comparable between genders, with a similar distribution across NRS categories. Despite this, women received analgesic treatment less frequently than men (62.0% vs. 66.9%; *p* < 0.001), whereas a greater effect is seen when particularly examining the use of opioid analgesics (50.0% vs. 57.1%; *p* < 0.001). This finding is consistent across all pain severity subgroups. Pain scores at patient handover were similar for women and men, indicating comparable pain levels at the end of prehospital care.

**Conclusions:**

Despite comparable pain intensity, women received analgesic and opioid treatment less frequently than men in prehospital care, indicating potential gender-based inequalities in pain management. These findings underscore the need for strategies to ensure equitable analgesic treatment.

**Supplementary Information:**

The online version contains supplementary material available at 10.1186/s12916-026-05131-0.

## Background

The adequate treatment of pain is a fundamental component of medical ethics and, particularly in emergency medical practice and represents one of the primary therapeutic goals [1]. Sex- and gender-specific differences in medical care have become an increasingly recognized topic in health research. Especially in pain management, studies have shown that women and men not only experience pain differently, but are being offered diverse treatment [2–4]. A recent study, addressing the frequency and intensity of pain therapy in emergency departments, revealed that pain in women was treated less frequently and less intensively [5]. In prehospital emergency medicine, where clinical assessment and decision making are to be made under pressure and in a time sparing manner, gender-based differences could unintentionally lead to disparities in care [6, 7]. However, there is a lack of systematic investigations into whether, and to what extent, a patient’s and an emergency physician’s gender influences pain management [6, 8].

The term “gender pain gap” originates from public health research and refers to differences in healthcare provision between men and women with chronic pain. These disparities are not limited to Germany, but have been observed across much of Europe in varying forms [9, 10]. Whether these differences can be extrapolated to the treatment of acute pain in emergencies remains uncertain and is the subject of this study.

The objective of the study is to systematically capture and analyze gender-specific difference in prehospital pain management by emergency physicians across various medical and trauma-related conditions. This study will examine whether male and female patients receive different care in terms of the implementation and intensity of pain treatment during emergency deployments.

## Methods

### Study design

This retrospective observational cohort study analyzed routine documentation from all primary helicopter emergency medical service (HEMS) missions conducted between January 2012 and June 2025 at all HEMS bases operated by DRF Stiftung Luftrettung (DRF, Filderstadt, Germany) across Germany. Each HEMS crew comprises at least one pilot, a HEMS Technical Crew Member (HEMS-TC) and an emergency physician. As an integral component of public emergency medical services (EMS), these helicopters are dispatched by regional emergency control centers alongside ground-based ambulance services for primary emergency response.

### Data processing

Operational data from HEMS missions are systematically recorded in a dedicated database. Mission data were documented using a standardized electronic form within the HEMSDER database system (Convexis, Germany). Since August 2022, documentation has been conducted using the NIDApad system (medDV GmbH, Fernwald, Germany). For the purposes of this study, the following parameters were extracted for analysis: patient age and gender, emergency physician gender, diagnosis, airway management, Numeric Rating Scale (NRS) scores at initial assessment and at patient handover, Glasgow Coma Scale (GCS) scores at initial assessment and at handover, and administration of pain medication.

**Fig. 1:**
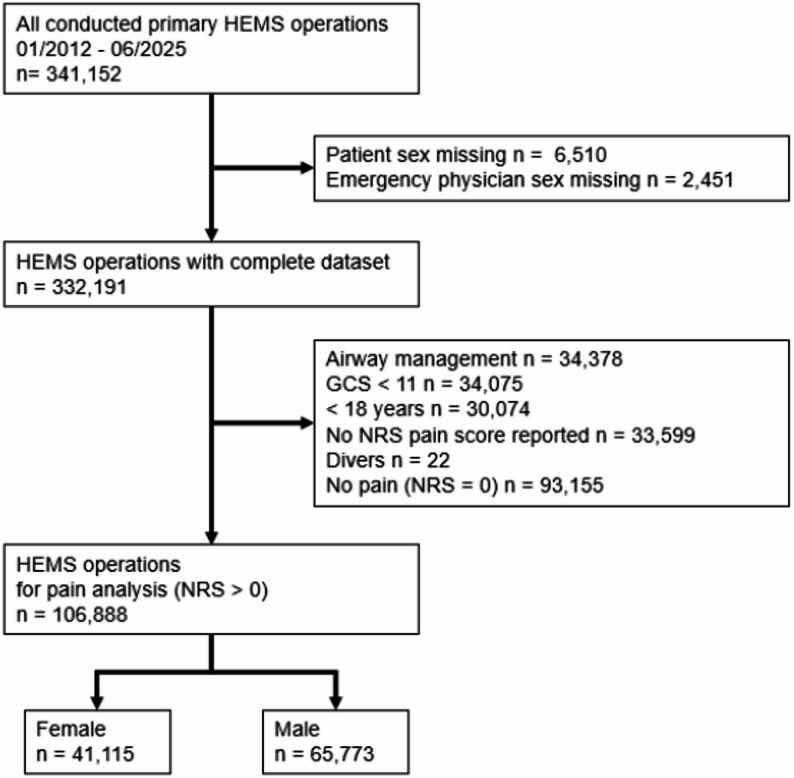
Study flow chart according to STROBE protocol

To ensure anonymization, a name-based algorithm was applied prior to data export to determine the gender of emergency physicians based on their first names. Patients who underwent airway management were not included in the study. Airway management procedures were categorized as follows: for the NIDA system (suctioning, intubation, preexisting intubation status, nasopharyngeal or oropharyngeal tube, and controlled ventilation) and for the HEMSDER system (endotracheal suctioning, controlled ventilation, endotracheal intubation, and other airway management). In accordance with the *Strengthening the Reporting of Observational Studies in Epidemiology* (STROBE) protocol, Fig. 1 depicts the exclusion process for patients with a GCS score < 11, those who received airway management, and individuals younger than 18 years [11]. Analgesic administration within the DRF Luftrettung HEMS system was guided by standardized internal protocols based on Numerical Rating Scale (NRS) assessment, consistent with national prehospital pain management guidelines. These protocols remained unchanged in their fundamental principles throughout the entire study period. For this study, the pain was classified according to established methods: no pain (NRS = 0), mild pain (NRS 1–3), moderate pain (NRS 4–6), and severe pain (NRS 7–10) [12]. The gender category “diverse” (n = 22) was excluded from the analysis. This group represented a negligible proportion of the sample (< 0.1%), and physician gender was recorded as male or female [10]. Taken all together, this investigation is limited to the socially predominant binary gender discrimination „man“ and „woman” to minimize confounding factors. We are fully aware of the existential data gap and the gender-based potential mistreatment that people outside this spectrum might suffer. Yet it is necessary to clearly distinguish between the examined subgroups. Assignment to the subgroups “man” and “woman” followed the gender designation on the patient’s identity documents without further investigation of biological sex traits. Surely, in this manner, there is also no tracing of chromosomal variations going alongside one or the other phenotype [13].

### Statistical analysis and ethical approval

Continuous variables were described and compared using mean and standard deviation. Nominal variables were analyzed using the Pearson Chi-Square Test. The Pearson correlation was applied for two-sided testing and effect sizes were evaluated with Cohen’s d for independent samples. A t-test for independent samples was conducted. All tests were considered significant for p values < 0.05. Analysis was conducted using IBM SPSS Statistics 29 (Armonk, NY, USA). Both documentation systems required the mandatory entry of core variables, including the NRS score, administered medication, and patient sex, at the point of care, ensuring the completeness of the primary dataset by design. Analyses were performed on complete cases; given the negligible rate of missing data in key variables, formal imputation was not applied.

The study was approved by the Ethics Committee of the Albert-Ludwigs-University Freiburg (No: 25-1272-S1, August 19th 2025) and registered with the German Register of Clinical Studies (No: DRKS00037729, August 19th 2025).

## Results

After exclusions as outlined in Fig. 1, a total of 106,888 cases were included in the analysis. Of these, 38.5% (*n* = 41,115) involved female patients and 61.5% (*n* = 65,773) male patients. Pain severity at initial assessment was comparable between women and men (4.94 ± 2.6 vs. 4.94 ± 2.6; *p* = 0.865) and showed a relatively even distribution across the three predefined pain categories. Specifically, NRS scores of 1–3 were observed in 36.4% of women and 36.0% of men, NRS 4–6 in 31.5% and 32.6%, and NRS 7–10 in 32.1% and 31.4%, respectively (Table 1). Binary logistic regression (Table 2) demonstrated that female patient sex was independently associated with a significantly lower likelihood of receiving opioid analgesia (OR = 0.905, 95% CI [0.881, 0.929], *p* < 0.001), after controlling for NACA score, trauma or non-trauma missions, patient age, and sex of the EMS provider. This finding indicates that the gender disparity in prehospital opioid administration persists as an independent effect.

**Table 1:** Characteristics (SD = Standard Deviation, NRS = Numeric Rating Scale)

	Female 38.5% (41,115)	Male 61.5% (65,773)	*p*-Value	Total 106,888
**Patient age (mean, SD)**	60.2 ± 21.3	53.8 ± 19.1	-	
**NACA (mean**,** SD)**	3,37 ± 0.82	3.56 ± 0.86	< 0.001	3.49 ± 0.85
**Any analgesia received (%**,** n) (**Fig. 2**)**	62.0% 25,480	66.9% 43,997	< 0.001	65.0% 69,477
**Opioids received (%**,** n) (**Fig. 2**)**	50.0% 20,556	57.1% 37,571	< 0.001	54.4% 58,127
**Initial NRS (mean**,** SD)**	4.94 ± 2.6	4.94 ± 2.6	0.865	4.94 ± 2.6
**Pain Group (Initial)**				
**Pain Group NRS 1–3**	36.4% 14,984	36.0% 23,682	-	36.2% 38,666
**Pain Group NRS 4–6**	31.5% 12,932	32.6% 21,447	-	32.2% 34,379
**Pain Group NRS 7–10**	32.1% 13,199	31.4% 20,644	-	31.7% 33,843
**Pain Group (Handover)**				
**No Pain**	13.5% 5,566	11.8% 7,733	-	11.5% 13,299
**Pain Group NRS 1–3**	72.6% 29,847	74.6% 49,048	-	73.8% 78,895
**Pain Group NRS 4–6**	12.7% 5,225	12.7% 8,334	-	12.7% 13,559
**Pain Group NRS 7–10**	1.2% 477	1.0% 658	-	1.1% 1,135
**Emergency Physician (EP)**				
**Female EP**	20.4% 8,383	20.0% 13,134	0.095	20.1% 21,517
**Male EP**	79.6% 32,732	80.0% 52,639	0.095	79.9% 85,371
**Any analgesia given by female EP (%**,** n) (**Fig. 2**)**	63.4% 5,315/8,383	68.6% 9,009/13,134	< 0.001	66.6% 14,324/21,517
**Any analgesia given by male EP (%**,** n) (**Fig. 2**)**	61.6% 20,165/32,732	66.5% 34,988/52,639	< 0.001	64.6% 55,153/85,371
**Diagnosis**				
**Traumatic**	51.4% 21,118	61.4% 40,353		57.5% 61,471
**Non-traumatic**	47.6 19,568	37.6% 24,708		41.4% 44,276
**Unknown**	1.0% 429	1.1% 712		1.1% 1,141

**Table 2:** Binary Logistic Regression Predicting Opioid Analgesic Administration (Step 1)

Variable	B	SE	*p*	OR	95% CI for OR	
					Lower	Upper
NACA score	0.562	0.008	< 0.001	1.754	1.726	1.783
Trauma mechanism	0.909	0.014	< 0.001	2.481	2.414	2.550
Patient age	−0.003	0.000	< 0.001	0.997	0.997	0.998
Sex of EMS provider (female vs. male)	0.089	0.016	< 0.001	1.093	1.059	1.128
Sex of patient (female vs. male)	−0.100	0.013	< 0.001	0.905	0.881	0.929
Constant	−2.117	0.038	< 0.001	0.120	—	—

Note. OR = odds ratio; CI = confidence interval; SE = standard error; NACA = National Advisory Committee for Aeronautics severity score. Variables entered in Step 1: NACA score, trauma mechanism, patient age, sex of EMS provider, sex of patient. Reference category for sex variables: female. Cut value = 0.500

**Fig. 2:**
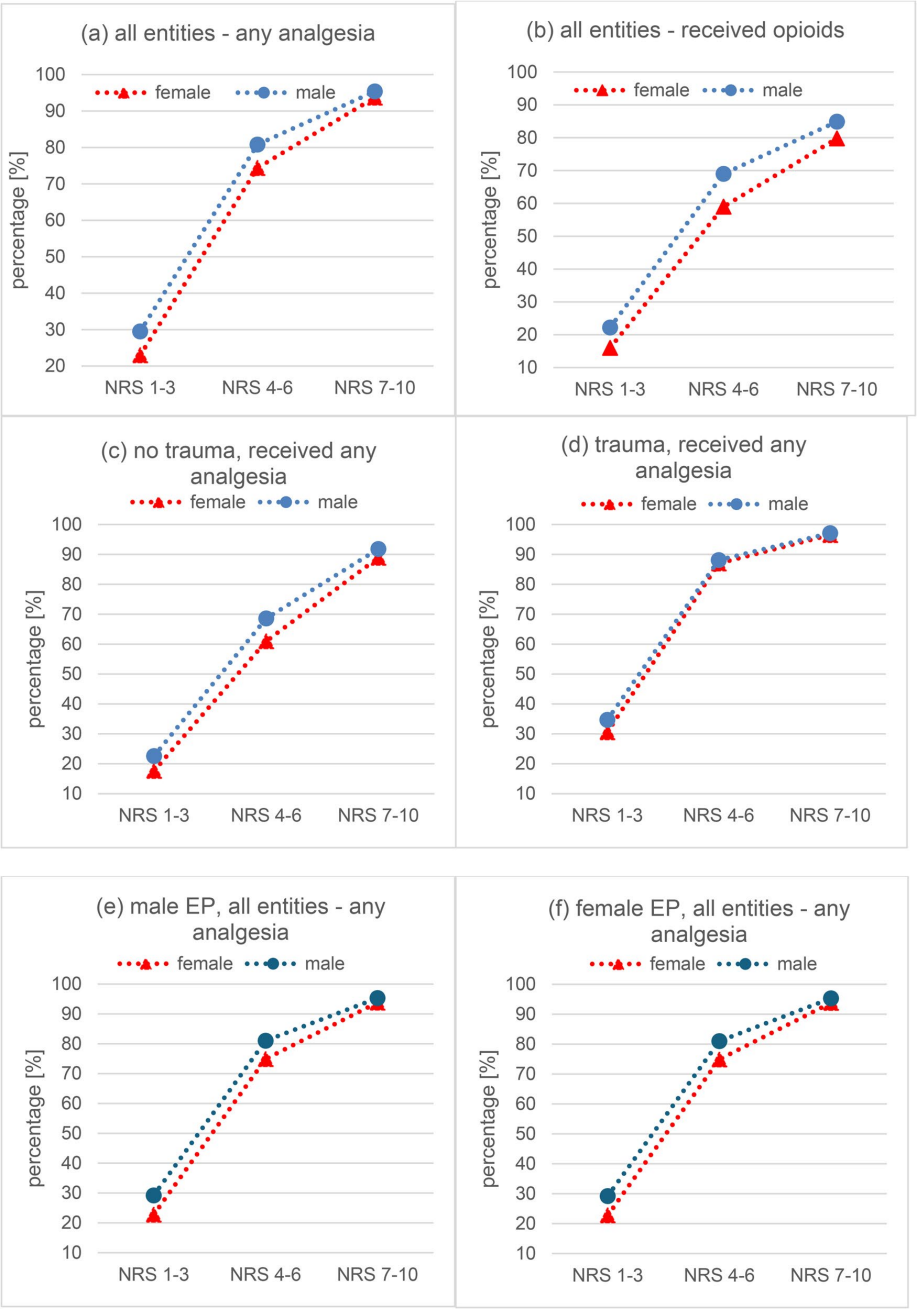
(**a**-**f**) The figures depict the proportion (%) of patients receiving analgesia stratified by pain severity (mild, moderate, and severe pain). Panel (**a**) shows the proportion receiving any analgesia across all entities, (**b**) opioid analgesia across all entities, (**c**) patients without trauma receiving any analgesia, and (**d**) trauma patients receiving any analgesia. Panels (**e**) and (**f**) present the proportion receiving any analgesia across all entities, stratified by male and female emergency physicians, respectively. All differences between female and male patients were statistically significant (*p* < 0.001) (EP = Emergency Physician, NRS = Numeric Rating Scale)

 Despite the presence of comparable baseline pain levels, analgesic management differed significantly between the two genders. In the overall population (NRS 1–10), analgesia was administered to 62.0% of women compared with 66.9% of men (*p* < 0.001). Opioid analgesia was provided to 50.0% of female patients, whereas 57.1% of male patients received opioid treatment (*p* < 0.001). This pattern of lower analgesic administration among women was consistently observed across all pain severity subgroups (Fig. 2). No differential treatment by gender of the emergency physician was observed; a 5% difference in analgesia treatment was observed between female and male physicians, with lower rates among female physicians (Table 1). 

**Fig. 3:**
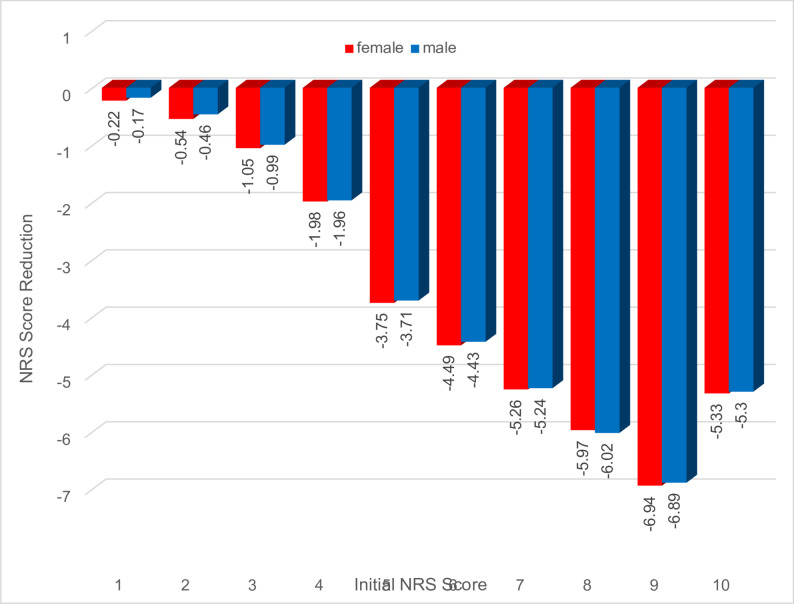
Reduction in pain intensity stratified by initial NRS score. Pain reduction was calculated as the difference between the NRS score at handover and the initial NRS score and is presented separately for female and male patients

At patient handover, pain scores again showed minimal differences between women and men, indicating comparable pain levels at the end of prehospital care (Table 1; Fig. 3). 

## Discussion

The findings showed comparable levels of reported pain between the two genders found within the prehospital HEMS setting. Yet fewer analgesics, particularly fewer opioids, were administered to female patients than to their male counterparts. This data contributes to the growing body of evidence demonstrating a persistent gender pain gap in acute care, where women’s pain is less aggressively treated even when severity is similarly documented [5, 14]. The discrepancy in the attending physician’s behavior was not being alleviated by his or her own gender. These results go along with various large data sets recorded in emergency departments or after hospital discharge, both of which settings also lack a sex concordance in treatment of pain and/or the prescription of opioids [5, 15]. Sex discordance has been associated with significantly worse outcomes for women in other medical settings [16].

While observing identical numeric pain scores between genders at initial evaluation and at handover, women were treated less with analgesics and specifically with opioids. In other words, the provider’s decision to — and how to — treat pain was associated with patient gender. Nonetheless, women’s documented NRS at handover showed the same reduction in perceived pain. This finding raises questions about previous statements attributing gender-based undertreatment primarily to differences in short-term responsiveness towards analgesics, although documentation bias, differential analgesic responsiveness, and nonpharmacological interventions represent alternative explanations for the comparable handover scores that cannot be excluded with the present data and are subject to further consideration within the Limitations Sects [5, 17, 18].

The results of the multivariate analysis confirm that the observed gender disparity in prehospital opioid administration is not merely a reflection of differences in mission severity or other patient characteristics such as age. Even after controlling for mission severity, trauma and non-trauma missions, patient age, and sex of the EMS provider, female patients remained significantly less likely to receive opioid analgesia. This independent effect suggests that the disparity cannot be attributed to female patients presenting with objectively less severe conditions or different mission profiles, but instead may reflect systematic differences in pain recognition and analgesic decision-making that are not explained by the clinical variables available in this registry-based dataset; given the retrospective observational design of this study, causal mechanisms cannot be established from these data. The magnitude of this effect, while statistically robust, remains modest (OR = 0.905), reflecting an approximately 10% reduction in the odds of opioid administration in female patients. In a cohort of this size, even effects of moderate clinical relevance reach high statistical precision, and the absolute effect at the level of an individual patient is small. Nonetheless, applied across the scale of nationwide HEMS operations, a consistent 10% reduction translates into a substantial number of women potentially receiving less analgesia than clinically required each year. This effect’s consistency across all subgroups, independent of mission severity, trauma status, and provider sex, underscores its practical significance.

The lack of association between emergency physician gender and the observed treatment gap supports the notion that implicit bias and structural factors are shared across providers, rather than being confined to one gender. This aligns with experimental vignette studies showing that both male and female clinicians tend to underestimate women’s pain and are less likely to recommend potent analgesia for female patients [5, 14].

### Context with existing literature

Multiple reviews and empirical studies have documented that women often receive less or delayed analgesia compared with men in emergency and procedural settings, even after adjustment for pain scores and clinical covariates. For example, large emergency department datasets from two countries demonstrated that female patients with painful conditions were significantly less likely to be prescribed any analgesic at discharge, whether opioid or non-opioid, than male patients with comparable reported pain [5, 14].

Prehospital data reflect similar patterns: when suffering from isolated extremity injury, women were less likely to receive prehospital analgesia than men and were less likely to receive morphine, despite similar or higher pain scores [19, 20]. However, some studies, which evaluated data from emergency departments, have reported no gender differences or even higher rates of analgesic use in women, emphasizing substantial heterogeneity across settings and highlighting the need for large, methodologically robust analyses such as the present HEMS study [21–23].

### Potential mechanisms underlying the gender pain gap

Several mechanisms may account for the reason why women receive fewer and less potent analgesics despite similar pain assessments:

- Stereotypes and implicit bias: Women’s pain is more frequently attributed to psychological or emotional causes, which can result in underestimation of symptom severity and more conservative pharmacologic treatment [14].
- Differential risk perception: Clinicians may overestimate the risk of opioid-related adverse events in women, such as nausea or respiratory depression, or may be more hesitant to use higher doses. This may be associated with a lower threshold for withholding opioids in female patients [17].
- Communication and documentation: Studies have shown that women’s pain scores and narratives are documented less consistently, and that women may need to report higher pain levels to receive equivalent treatment [5, 14].

The finding of no difference in NRS-adjusted pain reduction between genders in this cohort suggests that once treatment is initiated, titration is broadly similar. The primary inequity appears to lie in the decision to initiate and escalate analgesia, particularly opioids. These results interfere with a smaller study of 778 patients with higher rates of insufficient pain management among patients treated by a female physician [24].

The missing gender concordance of treatment behavior strengthens the overall impression of differing perspectives on pain, which depend on the perceived patient gender, and result being disadvantageous for women. The latter has a complex, long-standing history showing a downplaying of women’s pain [25]. The notion from multiple emergency departments describing higher analgesic or opioid use whenever the provider’s gender matched the patient’s gender [26] was not observed here, a pattern that corresponds with — though does not prove — structural rather than individual-provider factors underlying the disparity.

Although comparable NRS reductions at handover were documented across both sexes despite lower opioid administration rates in female patients (OR = 0.905), any decision to withhold or administer opioids must weigh the risk of adverse effects. Opioid-induced ventilatory impairment is among the most feared of these, as it does not strictly follow linear dose-dependency [27, 28]. Neither body mass index, sex, nor age alone significantly alters the risk of apnea/hypopnea [29]. A more cautious opioid threshold in female patients would not, by itself, explain this pattern; an alternative non-causal interpretation is that physicians underestimate pain more often in female patients. A small study of 77 emergency department patients found physician-documented pain has been underestimating patient pain in 70% of cases, this happening disproportionately often among women [30], a pattern echoed in studies of interactive pain assessment [31]. Taken together, these observations are compatible with the interpretation that opioid administration tracks more closely with physicians’ subjective assessment of pain than with patients’ ratings, although documentation bias and differences in analgesic responsiveness remain plausible alternative explanations that cannot be distinguished with the present data.

Given the acuity and time sensitivity of HEMS missions, systematic differences in analgesic use by patient gender are particularly concerning. Undertreatment of pain in female HEMS patients may contribute to avoidable suffering, impaired physiological stability, and a worse overall patient experience. The lack of modification by physician gender indicates that interventions should target systems and protocols, such as standardized pain-based analgesia algorithms and decision support, rather than focusing solely on individual provider characteristics. These findings support integrating explicit, gender-neutral pain management thresholds, such as NRS-based opioid titration protocols, into prehospital guidelines, coupled with feedback and audit on gender-stratified analgesia rates for HEMS teams. Some federal-state quality control authorities have already implemented gender-neutral quality indicators for prehospital pain therapy protocols [32].

Educational initiatives addressing implicit bias in pain assessment and management, anchored in data from large prehospital cohorts such as the present study, may help narrow the gender pain gap in this high-stakes environment.

Further studies investigating the gender pain gap in specific clinical presentations or its prevalence across different age groups, as well as the consistency of these results with those of ground-based emergency medical services, will be necessary to derive additional recommendations for action in daily practice.

### Limitations

A notable strength of this study lies in its heterogeneous cohort, combined with nationwide coverage and exceptionally high case numbers. However, a key limitation is the inherently subjective and inter-individual nature of pain assessment, which may introduce variability into the reported pain scores. In particular, the handover pain assessment may be influenced by the emergency physicians` desire to have achieved successful treatment. Furthermore, we cannot account for the effects on pain intensity that were potentially being modulated by the quality of the interindividual relationship between the patient and his or her medical provider. Communication-based pain reduction has been supported by substantial data from periprocedural observations [33–35]. It is yet to be determined to what extent communication per se follows a certain gender bias, and to what extent hypnotic communication is able to modulate pain intensity in outer hospital deployments. Additionally, comparable handover NRS scores in the context of lower opioid administration rates in female patients may reflect greater analgesic responsiveness, a ceiling effect in prehospital pain reduction, or a documentation bias resulting from physicians’ inclination to record scores indicative of successful treatment — all of which limit the interpretability of handover NRS values as an objective outcome measure. Taken together, these considerations preclude a straightforward causal interpretation of the comparable handover pain scores and do not diminish the clinical relevance of the observed systematic difference in analgesic administration between male and female patients.

Patients with severe clinical courses were excluded (e.g., those requiring airway management or presenting with a Glasgow Coma Scale score < 11). Consequently, this analysis is limited to conscious, adult patients with pain, and the findings may not be generalizable to critically ill, hemodynamically or respiratory unstable, or non-communicative patient populations. Provider-level characteristics beyond physician sex — including years of clinical experience, medical specialty background, and individual team composition — were unavailable in this registry-based dataset and therefore could not be included in the multivariate model.

## Conclusions

Based on the findings, potential treatment inequalities are identified. Despite comparable pain intensity at initial assessment and at patient handover, women received analgesic therapy less frequently than men. This discrepancy was particularly evident in the administration of opioid analgesia, suggesting a gender-based difference in pain management. These findings highlight the need to develop targeted strategies to ensure equitable pain treatment in prehospital care.

## Supplementary Information

Below is the link to the electronic supplementary material.

Supplementary Material 1: Additional File 1: Figures S1-S6

## Data Availability

The dataset analyzed in this study comprises sensitive pre-hospital patient data from helicopter emergency medical service (HEMS) operations, provided by DRF Luftrettung gGmbH under a restricted data-use agreement. Public deposition is not permissible due to: the classification of the data as special-category personal health data under Art. 9 GDPR and applicable German data protection legislation; the scope of patient consent; contractual restrictions imposed by the data owner; and applicable ethical requirements. Data may be made available to qualified researchers upon reasonable request. Requests should be directed to the Scientific Advisory Board (Wissenschaftlicher Arbeitskreis) of DRF Luftrettung gGmbH via WAK@drf-luftrettung.de. Access is subject to review and approval by the WAK- Board, execution of a formal data use agreement, and compliance with applicable data protection regulations. Any approved data use is restricted to the purposes specified in the access agreement; downstream redistribution and unrestricted secondary use are not permitted.

## Declarations

### Language check

During the preparation of this work the authors used Microsoft Word 365 Editor and Grammarly to check language and readability. AI contributed to this manuscript only by providing assisted copy editing. After using this service, the authors reviewed and edited the content as needed and take full responsibility for the content of the publication. The manuscript adheres to the journal’s guidelines for the use of AI.

## Abbreviations

EMS: Emergency Medical Service
GCS: Glasgow Coma Scale
HEMS: Helicopter Emergency Medical Service
HEMS-TC: HEMS Technical Crewmember
NACA: National Advisory Committee for Aeronautics (Score)
NRS: Numeric Rating Scale
STROBE: Strengthening the Reporting of Observational Studies in Epidemiology

## References

[1] Venkat A, Fromm C, Isaacs E, Ibarra J, Committee SE. An Ethical Framework for the Management of Pain in the Emergency Department. Acad Emerg Med. 2013;20:716–23. 10.1111/acem.12158. 23859586 10.1111/acem.12158

[2] Laughey W, Vincent K, Iyer S, Cobo MM, Slater R. Pain in women: bridging the gender pain gap. PAIN Rep. 2025;10:e1276. 10.1097/PR9.0000000000001276. 40401085 10.1097/PR9.0000000000001276 PMC12094398

[3] Weiss SJ, Ernst AA, Phillips J, Hill B. Gender differences in state-wide EMS transports. Am J Emerg Med. 2000;18:666–70. 10.1053/ajem.2000.16299. 11043618 10.1053/ajem.2000.16299

[4] Meisel ZF, Armstrong K, Crawford Mechem C, Shofer FS, Peacock N, Facenda K, et al. Influence of Sex on the Out-of-hospital Management of Chest Pain. Acad Emerg Med. 2010;17:80–7. 10.1111/j.1553-2712.2009.00618.x. 20078440 10.1111/j.1553-2712.2009.00618.x

[5] Guzikevits M, Gordon-Hecker T, Rekhtman D, Salameh S, Israel S, Shayo M, et al. Sex bias in pain management decisions. Proc Natl Acad Sci. 2024;121:e2401331121. 10.1073/pnas.2401331121. 39102546 10.1073/pnas.2401331121 PMC11331074

[6] Albrecht E, Taffe P, Yersin B, Schoettker P, Decosterd I, Hugli O. Undertreatment of acute pain (oligoanalgesia) and medical practice variation in prehospital analgesia of adult trauma patients: a 10 year retrospective study. BJA Br J Anaesth. 2013;110:96–106. 10.1093/bja/aes355. 23059961 10.1093/bja/aes355

[7] Lewis JF, Zeger SL, Li X, Mann NC, Newgard CD, Haynes S, et al. Gender Differences in the Quality of EMS Care Nationwide for Chest Pain and Out-of-Hospital Cardiac Arrest. Womens Health Issues. 2019;29:116–24. 10.1016/j.whi.2018.10.007. 30545703 10.1016/j.whi.2018.10.007

[8] Karas A, Fridrich L, Radomislensky I, Avital G, Gendler S, Chen J, et al. The Influence of Gender Bias: Is Pain Management in the Field Affected by Health Care Provider’s Gender? Prehospital Disaster Med. 2022;37:638–44. 10.1017/S1049023X2200111X. 35924723 10.1017/S1049023X2200111X

[9] Bimpong K, Thomson K, Mcnamara CL, Balaj M, Akhter N, Bambra C, et al. The Gender Pain Gap: gender inequalities in pain across 19 European countries. Scand J Public Health. 2022;50:287–94. 10.1177/1403494820987466. 33568013 10.1177/1403494820987466 PMC8873965

[10] Heidari S, Babor TF, De Castro P, Tort S, Curno M. Sex and Gender Equity in Research: rationale for the SAGER guidelines and recommended use. Res Integr Peer Rev. 2016;1:2. 10.1186/s41073-016-0007-6. 29451543 10.1186/s41073-016-0007-6 PMC5793986

[11] von Elm E, Altman DG, Egger M, Pocock SJ, Gøtzsche PC, Vandenbroucke JP. The Strengthening the Reporting of Observational Studies in Epidemiology (STROBE) statement: guidelines for reporting observational studies. Lancet. 2007;370:1453–7. 10.1016/S0140-6736(07)61602-X. 18064739 10.1016/S0140-6736(07)61602-X

[12] Karcioglu O, Topacoglu H, Dikme O, Dikme O. A systematic review of the pain scales in adults: Which to use? Am J Emerg Med. 2018;36:707–14. 10.1016/j.ajem.2018.01.008. 29321111 10.1016/j.ajem.2018.01.008

[13] Kaufman MR, Eschliman EL, Karver TS. Differentiating sex and gender in health research to achieve gender equity. Bull World Health Organ. 2023;101:666–71. 10.2471/BLT.22.289310. 37772198 10.2471/BLT.22.289310 PMC10523819

[14] Grinberg K, Sela Y. A Literature Review on Pain Management in Women During Medical Procedures: Gaps, Challenges, and Recommendations. Med (Mex). 2025;61:1352. 10.3390/medicina61081352. 10.3390/medicina61081352 PMC12388214 40870397

[15] Rambachan A, Joshi M, Auerbach AD, Fang MC. Sex concordance between physicians and patients and discharge opioid prescribing. J Hosp Med. 2024;19:605–9. 10.1002/jhm.13389. 38721898 10.1002/jhm.13389 PMC11222022

[16] Wallis CJD, Jerath A, Coburn N, Klaassen Z, Luckenbaugh AN, Magee DE, et al. Association of Surgeon-Patient Sex Concordance With Postoperative Outcomes. JAMA Surg. 2022;157:146–56. 10.1001/jamasurg.2021.6339. 34878511 10.1001/jamasurg.2021.6339 PMC8655669

[17] LeResche L. Defining Gender Disparities in Pain Management. Clin Orthop. 2011;469:1871–7. 10.1007/s11999-010-1759-9. 21210309 10.1007/s11999-010-1759-9 PMC3111774

[18] Lord B, Cui J, Kelly A-M. The impact of patient sex on paramedic pain management in the prehospital setting. Am J Emerg Med. 2009;27:525–9. 10.1016/j.ajem.2008.04.003. 19497456 10.1016/j.ajem.2008.04.003

[19] Raftery KA, Smith-Coggins R, Chen AH. Gender-Associated Differences in Emergency Department Pain Management. Ann Emerg Med. 1995;26:414–21. 10.1016/S0196-0644(95)70107-9. 7574121 10.1016/s0196-0644(95)70107-9

[20] Michael GE, Sporer KA, Youngblood GM. Women are less likely than men to receive prehospital analgesia for isolated extremity injuries. Am J Emerg Med. 2007;25:901–6. 10.1016/j.ajem.2007.02.001. 17920974 10.1016/j.ajem.2007.02.001

[21] Hornik ES, Thode HC, Singer AJ. Analgesic use in ED patients with long-bone fractures: A national assessment of racial and ethnic disparities. Am J Emerg Med. 2023;69:11–6. 10.1016/j.ajem.2023.03.054. 37027957 10.1016/j.ajem.2023.03.054

[22] Lau T, Hayward J, Vatanpour S, Innes G. Sex-related differences in opioid administration in the emergency department: a population-based study. Emerg Med J EMJ. 2021;38:467–73. 10.1136/emermed-2020-210215. 33853938 10.1136/emermed-2020-210215

[23] Almutairi A, Coyer F, Keogh S, Hughes J. Factors influencing pain management in patients presenting to the emergency department: A mixed-method systematic review. Int J Nurs Stud. 2025;172:105214. 10.1016/j.ijnurstu.2025.105214. 40992019 10.1016/j.ijnurstu.2025.105214

[24] Oberholzer N, Kaserer A, Albrecht R, Seifert B, Tissi M, Spahn DR, et al. Factors Influencing Quality of Pain Management in a Physician Staffed Helicopter Emergency Medical Service. Anesth Analg. 2017;125:200. 10.1213/ANE.0000000000002016. 28489643 10.1213/ANE.0000000000002016

[25] Hoffmann DE, Tarzian AJ. The girl who cried pain: a bias against women in the treatment of pain. J Law Med Ethics J Am Soc Law Med Ethics. 2001;29:13–27. 10.1111/j.1748-720x.2001.tb00037.x. 10.1111/j.1748-720x.2001.tb00037.x 11521267

[26] Safdar B, Heins A, Homel P, Miner J, Neighbor M, DeSandre P, et al. Impact of physician and patient gender on pain management in the emergency department–a multicenter study. Pain Med Malden Mass. 2009;10:364–72. 10.1111/j.1526-4637.2008.00524.x. 10.1111/j.1526-4637.2008.00524.x 18992042

[27] Macintyre PE, Loadsman JA, Scott DA. Opioids, ventilation and acute pain management. Anaesth Intensive Care. 2011;39:545–58. 10.1177/0310057X1103900405. 21823370 10.1177/0310057X1103900405

[28] Pattullo GG. Clinical implications of opioid-induced ventilatory impairment. Anaesth Intensive Care. 2022;50:52–67. 10.1177/0310057X211070292. 35189729 10.1177/0310057X211070292

[29] Schwarzer A, Aichinger-Hinterhofer M, Maier C, Vollert J, Walther JW. Sleep-disordered breathing decreases after opioid withdrawal: results of a prospective controlled trial. Pain. 2015;156:2167–74. 10.1097/j.pain.0000000000000279. 26121253 10.1097/j.pain.0000000000000279

[30] Alotaibi M, Aljahany M, Alhamdan Z, Alsaffar M, Almojally A, Alassaf W. Differences in acute pain perception between patients and physicians in the emergency department. Heliyon. 2022;8:e11462. 10.1016/j.heliyon.2022.e11462. 36406726 10.1016/j.heliyon.2022.e11462 PMC9667246

[31] Ruben MA, Stosic MD. Documenting Race and Gender Biases in Pain Assessment and a Novel Intervention Designed to Reduce Biases. J Pain. 2024;25:104550. 10.1016/j.jpain.2024.104550. 38692397 10.1016/j.jpain.2024.104550 PMC11793930

[32] Indikatoren - Datenblätter | SQR-BW. https://www.sqrbw.de/indikatoren/datenblaetter. Accessed 28 Dec 2025.

[33] Fusco N, Bernard F, Roelants F, Watremez C, Musellec H, Laviolle B, et al. Hypnosis and communication reduce pain and anxiety in peripheral intravenous cannulation: Effect of Language and Confusion on Pain During Peripheral Intravenous Catheterization (KTHYPE), a multicentre randomised trial. Br J Anaesth. 2020;124:292–8. 10.1016/j.bja.2019.11.020. 31862159 10.1016/j.bja.2019.11.020

[34] Nowak H, Zech N, Asmussen S, Rahmel T, Tryba M, Oprea G, et al. Effect of therapeutic suggestions during general anaesthesia on postoperative pain and opioid use: multicentre randomised controlled trial. BMJ. 2020;371:m4284. 10.1136/bmj.m4284. 33303476 10.1136/bmj.m4284 PMC7726311

[35] Schmutz T, Iglesias K, Peier F, Ribordy V, Donner V, Magnin J-L, et al. Effect of hypnotic communication on pain during arterial blood gas standardized procedures in the emergency department compared with traditional communication: a triple-blind randomized controlled trial (POPAIN study). Eur J Emerg Med Off J Eur Soc Emerg Med. 2025. 10.1097/MEJ.0000000000001292. 10.1097/MEJ.0000000000001292 PMC13124270 41258671

