# Supplementary material for: The gender pain gap in prehospital analgesia: the role of patient and provider gender – an analysis of 106,888 helicopter emergency medical service (HEMS) missions
Source: BMC Med. 2026 Aug 6;24:426. doi: 10.1186/s12916-026-05131-0 (PMC13453089; doi:10.1186/s12916-026-05131-0)
Supplement: Supplementary file 1 [file 12916_2026_5131_MOESM1_ESM.docx]

**Supplement**

no trauma, received opioids

percentage [%]

|  | female | male |
| --- | --- | --- |
| NRS 1-3 | 11.7 | 16.9 |
| NRS 4-6 | 44.6 | 57 |
| NRS 7-10 | 73 | 82 |

trauma, received opioids

percentage [%]

|  | female | male |
| --- | --- | --- |
| NRS 1-3 | 21.8 | 26.3 |
| NRS 4-6 | 71.5 | 76 |
| NRS 7-10 | 83.5 | 86.1 |

**FigS1** Proportion (%) of patients receiving opioid analgesia stratified by pain severity (mild, moderate, and severe pain) for no-trauma and trauma entities

male EP, all entities - opioids

percentage [%]

|  | female | male |
| --- | --- | --- |
| NRS 1-3 | 15.8 | 22 |
| NRS 4-6 | 59.1 | 69.4 |
| NRS 7-10 | 80.2 | 84.7 |

female EP, all entities - opioids

percentage [%]

|  | female | male |
| --- | --- | --- |
| NRS 1-3 | 16.5 | 23.2 |
| NRS 4-6 | 58.6 | 67.4 |
| NRS 7-10 | 78.4 | 85.5 |

**FigS2** Proportion (%) of patients receiving analgesia stratified by pain severity (mild, moderate, and severe pain) for male and female emergency physicians (EP)

no trauma, male EP - any analgesia

percentage [%]

|  | female | male |
| --- | --- | --- |
| NRS 1-3 | 17.7 | 22.4 |
| NRS 4-6 | 60.8 | 68.9 |
| NRS 7-10 | 89 | 91.5 |

no trauma female EP - any analgesia

percentage [%]

|  | female | male |
| --- | --- | --- |
| NRS 1-3 | 17.7 | 23.8 |
| NRS 4-6 | 62.1 | 67.2 |
| NRS 7-10 | 88.4 | 92.4 |

**FigS3** Proportion (%) of patients receiving any analgesia stratified by pain severity (mild, moderate, and severe pain) for no-trauma entities and male and female EPs

no trauma male EP - opioids

percentage [%]

|  | female | male |
| --- | --- | --- |
| NRS 1-3 | 11.7 | 16.6 |
| NRS 4-6 | 44.1 | 57.2 |
| NRS 7-10 | 73.7 | 81.7 |

no trauma female EP - opioids

percentage [%]

|  | female | male |
| --- | --- | --- |
| NRS 1-3 | 11.6 | 17.8 |
| NRS 4-6 | 46.5 | 55.9 |
| NRS 7-10 | 72 | 82.9 |

**FigS4** Proportion (%) of patients receiving opioids stratified by pain severity (mild, moderate, and severe pain) for no-trauma entities and male and female EPs

trauma, male EP - any analgesia

percentage [%]

|  | female | male |
| --- | --- | --- |
| NRS 1-3 | 30.1 | 34.6 |
| NRS 4-6 | 87.4 | 88.8 |
| NRS 7-10 | 96.6 | 97.1 |

trauma, female EP - any analgesia

percentage [%]

|  | female | male |
| --- | --- | --- |
| NRS 1-3 | 32.4 | 35.2 |
| NRS 4-6 | 85.5 | 86.6 |
| NRS 7-10 | 96.1 | 97.2 |

**FigS5** Proportion (%) of patients receiving any analgesia stratified by pain severity (mild, moderate, and severe pain) for trauma entities and male and female EPs

trauma, male EP - opioids

percentage [%]

|  | female | male |
| --- | --- | --- |
| NRS 1-3 | 21.5 | 26 |
| NRS 4-6 | 72 | 76.4 |
| NRS 7-10 | 83.8 | 86 |

trauma, female EP - opioids

percentage [%]

|  | female | male |
| --- | --- | --- |
| NRS 1-3 | 22.9 | 27.4 |
| NRS 4-6 | 69.6 | 74.3 |
| NRS 7-10 | 82.3 | 86.7 |

**FigS6** Proportion (%) of patients receiving opioids stratified by pain severity (mild, moderate, and severe pain) for trauma entities and male and female EPs

**Supplement 1:** The figures above depict the proportion (%) of patients receiving analgesia stratified by pain severity (mild, moderate, and severe pain). The panels show the combinations of entities, medication, and gender of emergency physicians. All differences between female and male patients were statistically significant (p < 0.001). (EP = Emergency Physician, NRS = Numeric Rating Scale).
